# Analyzing the Costs and Benefits of Brief Intervention

**Published:** 2006

**Authors:** Marlon P. Mundt

**Affiliations:** Marlon P. Mundt, M.S., is a senior programmer analyst with the Department of Family Medicine at the University of Wisconsin–Madison, Madison, Wisconsin

**Keywords:** health services research, primary care facility, problematic AOD (alcohol and other drug) use, social and economic cost of AOD, treatment cost, AOD use screening method, intervention, cost-effectiveness of AOD health services, cost-benefit analysis, Trial for Early Alcohol Treatment (Project TrEAT)

## Abstract

The Trial for Early Alcohol Treatment, Project TrEAT, was a randomized controlled trial of screening and brief intervention in primary care clinics. One of the few such trials to be analyzed in terms of cost-effectiveness, Project TrEAT was examined from two perspectives. The analysis from the perspective of medical care providers focused on clinic and hospital costs, contrasting the benefits that directly reduced medical expenditures with the costs to providers. The analysis from the societal perspective took all of the intervention’s costs and benefits into account. Both components of this study revealed that Project TrEAT led to a reduction in alcohol consumption by high-risk drinkers and a corresponding reduction in medical and societal costs. Overall, this study supported the cost-effectiveness of Project TrEAT, concluding that its costs were outweighed by its benefits.

The effectiveness of screening and brief intervention for alcohol problems has been demonstrated in a variety of settings (Moyer et al. 2002), but the cost-effectiveness of brief intervention is less well studied. One of the few randomized trials in which the economic costs and benefits of brief intervention have been analyzed is the Trial for Early Alcohol Treatment, or Project TrEAT (Fleming et al. 1997). The economic analysis of this project is described in this section (for a summary of the analysis, see the accompanying table).

Project TrEAT was a randomized controlled trial of screening and brief intervention in primary care clinics. The intervention consisted of two 15-minute sessions with a physician, 4 weeks apart, and a followup call with a clinic nurse 2 weeks after each meeting with the physician, for a total of four contacts. The 12-month and 48-month followup results on the economic costs and benefits of the study have been presented in two reports (Fleming et al. 2000, 2002).

## Benefits

This intervention resulted in economic benefits in the form of reduced hospital and emergency department use, fewer criminal and legal events, and fewer motor vehicle incidents (crashes, violations, and related arrests). Both the 12-month and the 48-month followups revealed cost–benefits of using the intervention.

In the first 12 months, intervention subjects reported fewer emergency department visits (107 visits by the 392 intervention subjects compared with 132 visits by the 382 control subjects) and fewer days of hospitalization (126 vs. 326). Emergency and hospital care was estimated to cost $421 per intervention subject compared with $943 per control subject, for a $522 cost differential in the first 12 months after intervention. Over 48 months, the cost differential in emergency and hospital care widened to $712 per patient.

Information on the medical care use outcomes of Project TrEAT relied on patient self-report, but data on legal and motor vehicle–related events were collected from government sources. The Wisconsin Department of Justice and the Wisconsin Department of Transportation performed record searches for all enrolled patients (Wisconsin Department of Justice 1994; Wisconsin Department of Transportation 1994). Consistency across multiple sources of data is a key consideration in determining the strength of the intervention, and the results from Project TrEAT demonstrated this consistency.

Legal outcomes—such as assault, child abuse, disorderly conduct, or criminal property damage—are rare events. The number of these events did not attain statistical significance over the study’s 48-month followup period but did follow the trend toward fewer events in the intervention group than the control group (28 arrests vs. 41). After applying event costs to each of the legal outcomes (Miller et al. 1996), the cost differential in favor of intervention was $102 per patient.

Motor vehicle crashes and violations also are relatively rare events, but at 48 months a statistically significant difference could be seen in favor of the intervention group. Two motor vehicle–related fatalities occurred in the control group (compared with none in the intervention group), as well as 55 percent more crashes involving injury (31 vs. 20) and 7 percent more collisions involving property damage only (72 vs. 67). Applying average costs per event (Miller et al. 1998) yielded a total benefit of $7,171 per patient in reduced motor vehicle costs.

In summary, each of the economic components of the analysis showed that the intervention led to positive outcomes at both 12 months and 48 months of followup. In the short term, most of the benefit could be seen in terms of health care use. It is possible that because legal and motor vehicle incidents occur rarely, a longer period of time must elapse before a reduction in the number of these events can be detected.

## Costs

The costs of the intervention protocol for Project TrEAT can be analyzed in terms of the following activities: staff training, screening for problem alcohol use, assessment of subjects’ appropriateness for intervention, the intervention itself, followup, and patient time and travel costs. The total cost of the intervention was estimated at $205 per intervention patient, broken down as $88 in screening and assessment costs, $23 in training costs, $55 in intervention costs, and $39 in patient time and travel costs (Fleming et al. 2000).

Screening and assessment account for more than 50 percent of the clinical costs of the intervention. Although the cost of an individual quantity-and-frequency screen of recent alcohol consumption is relatively small, screening all of a clinic’s patients can consume considerable resources. The impact of the intervention depends on the prevalence of problem drinking in the given population. In Project TrEAT, 17 percent of patients scored positive in the initial screen, but 4 percent were ultimately randomized to the trial. Although some of this discrepancy was attributable to the more sensitive data collection instruments used later in the assessment phase, which indicated that some of the patients actually did not meet the specified criteria for problem drinking, some of the difference between the percentage of patients who initially tested positive and the percentage who ultimately participated in the trial also may be attributable to patients’ reluctance to be involved in a research study. Thus, the true percentage of patients for whom brief intervention was appropriate is likely to have been somewhere between 4 percent and 17 percent. Small changes in the percentage of patients included in a trial can have a considerable impact on the ultimate cost. If the participation rate had been 6 percent, the screening cost per intervention subject would have been reduced by 28 percent (from $88 to $64 per intervention patient).

Project TrEAT was carried out in 17 clinics and involved 64 family physicians and general internists. The cost estimates were based on hourly wages of the staff involved in the various stages of the intervention, but in practice, this cost could vary depending on the type of staff performing the screening and intervention. Some clinics may have staff dedicated to the type of counseling employed in this project, so training costs could be overstated. The economic analysis included costs for training the study physicians, but training costs were minimal after the initial phase of the project. Also, the direct economic effect on the clinic of implementing the intervention protocol could have been mitigated because some or all of the intervention costs may have been reimbursable.

An essential feature of the economic analysis of Project TrEAT was the inclusion of patient costs. Patient willingness to participate depends on time and travel costs as well as perceived benefit. In settings where there is considerable variation in cost to the patient, the generalizability of this study’s results could vary.

**Table:** Costs and Benefits of Project TrEAT

*Benefits*					
Hospital & Emergency Dept. (ED) Use	No. ED Visits	No. Days Hospitalized	ED & Hospital Costs		
(at 12 months)	(at 12 months)	(12 mos.)		(48 mos.)
Intervention Subjects	107	126	$421		$1,394
Control Subjects	132	326	$943		$2,106
**Legal Outcomes**	**No. Arrests**	**Event Costs**			

Intervention Subjects	28	$269			
Control Subjects	41	$371			
**Motor Vehicle Outcomes**	**Fatalities**	**Crashes With Injuries**	**Crashes With Property Damage Only**		**Average Costs of Incidents**

Intervention Subjects	0	20	67		$ 3,839
Control Subjects	2	31	72		$11,010
***Costs***					
	**Screening & Assessment**	**Staff Training**	**Intervention**	**Patient Time & Travel**	**Total**

Per Intervention Subject	$88	$23	$55	$39	$205

## Conclusion

To analyze the economic results of Project TrEAT, two benefit–cost ratios were calculated. The first ratio reflected benefits and costs from the perspective of the medical care provider and included only the clinic and hospital costs. In this case, only the benefits that directly reduced medical expenditures were contrasted to provider costs. The benefit–cost ratio from the medical perspective was 4.3 to 1 (95-percent confidence interval: [0.6, 8.0]).

The second perspective from which the intervention could be considered—the societal perspective—considered all costs and benefits. After 48 months of followup, the estimated societal benefit–cost ratio was 39 to 1. Although this ratio is much larger than the medical perspective ratio, it depends on rare, high-cost events, such as traffic fatalities, so the 95-percent confidence interval for the ratio is quite large, from a minimum of 5.4 up to a maximum of 72.5.

In summary, the economic analysis of the Project TrEAT data supports the cost-effectiveness of brief intervention. The reduction in drinking levels among the high-risk drinkers in the study appears to be coupled with a corresponding reduction in medical and societal costs. These benefits are sufficient to outweigh the cost of the intervention.

## References

[b1-34-36] Fleming MF, Barry KL, Manwell LB (1997). Brief physician advice for problem alcohol drinkers: A randomized controlled trial in community-based primary care practices. JAMA: Journal of the American Medical Association.

[b2-34-36] Fleming MF, Mundt MP, French MT (2000). Benefit-cost analysis of brief physician advice with problem drinkers in primary care settings. Medical Care.

[b3-34-36] Fleming MF, Mundt MP, French MT (2002). Brief physician advice for problem drinkers: Long-term efficacy and benefit-cost analysis. Alcoholism: Clinical and Experimental Research.

[b4-34-36] Miller TR, Cohen MA, Wiersema B (1996). Victim Costs and Consequences: A New Look. National Institute of Justice Research Report.

[b5-34-36] Miller TR, Lestina DC, Spicer RS (1998). Highway crash costs in the United States by driver age, blood alcohol level, victim age, and restraint use. Accident Analysis & Prevention.

[b6-34-36] Moyer A, Finney JW, Swearingen CE, Vergun P (2002). Brief interventions for alcohol problems: A meta-analytic review of controlled investigations in treatment-seeking and non-treatment-seeking populations. Addiction.

[b7-34-36] Wisconsin Department of Justice, Crime Information Bureau, Record Check Unit (1994). Electronic record search for criminal records.

[b8-34-36] Wisconsin Department of Transportation (1994). Electronic search for accident and traffic violation records.

